# Use of *Streptococcus salivarius* K12 in supporting the mucosal immune function of active young subjects: A randomised double-blind study

**DOI:** 10.3389/fimmu.2023.1129060

**Published:** 2023-03-02

**Authors:** Alexander Bertuccioli, Marco Gervasi, Giosuè Annibalini, Beatrice Binato, Fabrizio Perroni, Marco B. L. Rocchi, Davide Sisti, Stefano Amatori

**Affiliations:** Department of Biomolecular Sciences, University of Urbino Carlo Bo, Urbino, Italy

**Keywords:** bacteriocin, IgA, *Streptococcus salivarius* K12, URTI, HIT

## Abstract

**Introduction:**

Upper respiratory tract infections (URTI) are the most common illnesses affecting athletes, causing absences from training and competition. Salivary immunoglobulin A (sIgA) is the main immune factor in saliva, and a consistent association between low concentrations of sIgA and an increased incidence of URTIs has been reported. The oral probiotic *Streptococcus salivarius* K12 has been suggested to have the potential to improve oral diseases and mucosal barrier function. However, the effects of this probiotic on active young subjects performing a high-intensity training (HIT) program have not been investigated.

**Methods:**

Active young students were randomised into a treated group (*S. salivarius* K12) and a control (placebo) group and asked to take the product daily for 30 days. After this period, participants performed a graded exercise test and five HIT sessions, all within 3 days. They were also asked to complete the Wisconsin Upper Respiratory Symptom Survey daily to monitor URTI’s presence. Before and after the 30 days, and at 0h, 24h and 72h after the last training session, saliva samples were collected to quantify sIgA level, secretion rate, and flow. The effect of *S. salivarius* K12 intake on these parameters was tested using an ANOVA for repeated measures.

**Results:**

Twenty (M = 14, F = 6) young subjects (23.5 ± 2.3 years old) participated in the study. The total accumulated training load (sRPE) in the supplementation period was similar in the two groups (treated: 4345 ± 3441 AU; control: 4969 ± 4165 AU; p > 0.05). Considering both sIgA level and secretion rate, significant time (F_(4,15)_ = 3.38; p = 0.037; F_(4,15)_ = 6.00; p = 0.004) and time×group interactions (F_(4,15)_ = 2.49; p = 0.049; F_(4,15)_ = 5.01; p = 0.009) were reported, with the treated group showing higher sIgA levels at 72h post-exercise and increased secretion rate both at 0h and 72h. The number of URTI episodes was similar in the treated and control groups (χ² = 1.83; p > 0.05).

**Conclusion:**

This study demonstrates that relatively short-term *S. salivarius* K12 supplementation increased sIgA level and secretion in healthy subjects performing a demanding exercise-training programme composed of HIT sessions.

## 1 Introduction

Exercise stimulates an immune response, dependent on the intensity, duration, and type of the stimulus (1–3). The athletes’ immune response capacity depends on parameters such as gender, age, clinical condition, and nutritional and training status (4, 5). However, high-intensity exercises may impair immune function and increase susceptibility to infection even in apparently healthy athletes (6). Exercise is defined as high intensity when it exceeds 70% of VO_2max_ (7). High-intensity interval training (HIT) is typically accomplished through intervals, as opposed to moderate-intensity continuous training, which is characterised by extended, continuous activity. HIT is characterised by a series of short (10 seconds) to long (5 minutes) intervals carried out at an intensity higher than the anaerobic threshold (7). HIT may temporarily perturb the immune system, triggering a decrease in immunological activity (8).

Upper respiratory tract infections (URTIs), including nose, sinuses and pharyngeals (9), are very common in athletes undergoing intense training, such as elite athletes (10, 11), which generally show a 2-6 times higher risk of URTI following exposure to the pathogen and/or environmental factors compared to non-athletes (12, 13). Intense training is acutely correlated with a reduction of several cellular and humoral elements characteristic of the immune response, including absolute salivary secretory immunoglobulin A (sIgA) concentration and secretory rate of sIgA (8, 14). This acute response seems to support the “open window” theory, the increased susceptibility to opportunistic infections from 3 to 72 hours following intense exercise (15, 16).

In the last years, oral probiotics’ use to promote oral health, salivary flow rate and sIgA contents gained increasing attention due to the possibility of preventing dental caries (17) and oral episodes of streptococcal and tonsillitis pharyngeal infections (18). This approach, based on a precision probiotics strategy (19), has also been defined as “bacterial therapy” or “bioprotic therapy” (18). A relevant example is the *Streptococcus salivarius* K12 microorganism isolated from the mouth of a healthy child (20). *S. salivarius* K12 is able to inhibit the growth of strains of *Streptococcus pyogenes b-haemolytic* (Lancefield group A, a common cause of tonsillitis and bacterial pharyngitis) (21, 22) and those of other pathogenic bacteria such as *Micrococcus luteus, Streptococcus anginosus, Eubacterium saburreum, Micromonas micros, Streptococcus pneumonia, Haemophilus influenzae* and *Moraxella catarrhalis* (23–25). This action is mainly due to lantibiotic cationic peptides *Salivaricin* A2 and *Salivaricin* B, encoded by the 190 kb megaplasmid found in the K12 strain (26). Notably, the *S. salivarius* K12 strain showed persistent colonisation of different upper respiratory tract tissues in newborns using the oral probiotic *S. salivarius* K12 (27, 28). Despite this recent evidence, the role of *S. salivarius* K12 as a precision probiotic to counteract athletes’ risk of developing URTI is still largely unknown.

In this study, twenty physically active subjects were randomised to receive either the *S. salivarius* K12 or placebo for 30 days and then performed a VO_2max_ test and five HIT sessions within three days. The absolute level and secretion rate of sIgA and URTI episodes were analysed throughout the study. Here, we hypothesised that the prophylactic administration of the *S. salivarius* K12 might increase the salivary flow rate and sIgA levels and reduce URTI episodes in the treated group compared to the placebo group.

## 2 Materials and methods

### 2.1 Participants

Participants were recruited from students at the University of Urbino Carlo Bo, Italy. Inclusion criteria were: age between 20 and 25 years; being in good health with no chronic disease; had not taken supplements at least three months before the start of the study; having practised physical activity at least three times a week in the last year. Participants signed a written informed consent before the study. The power analysis was performed considering the repeated measures ANOVA test: considering time as a repeated measure (five measures) and group membership as a binary between factor, with a cautionary non-sphericity correction coefficient = 0.8, with effect size *f* = 0.68, alfa = 0.05 and 1-beta = 0.8, a total of 24 subjects must be enrolled (29). The study was conducted according to the principles stated in the Declaration of Helsinki, and it was approved by the Ethics Committee for Human Experimentation of Urbino University Carlo Bo (no. of approval 29_2020).

### 2.2 Experimental design

**Figure 1:**
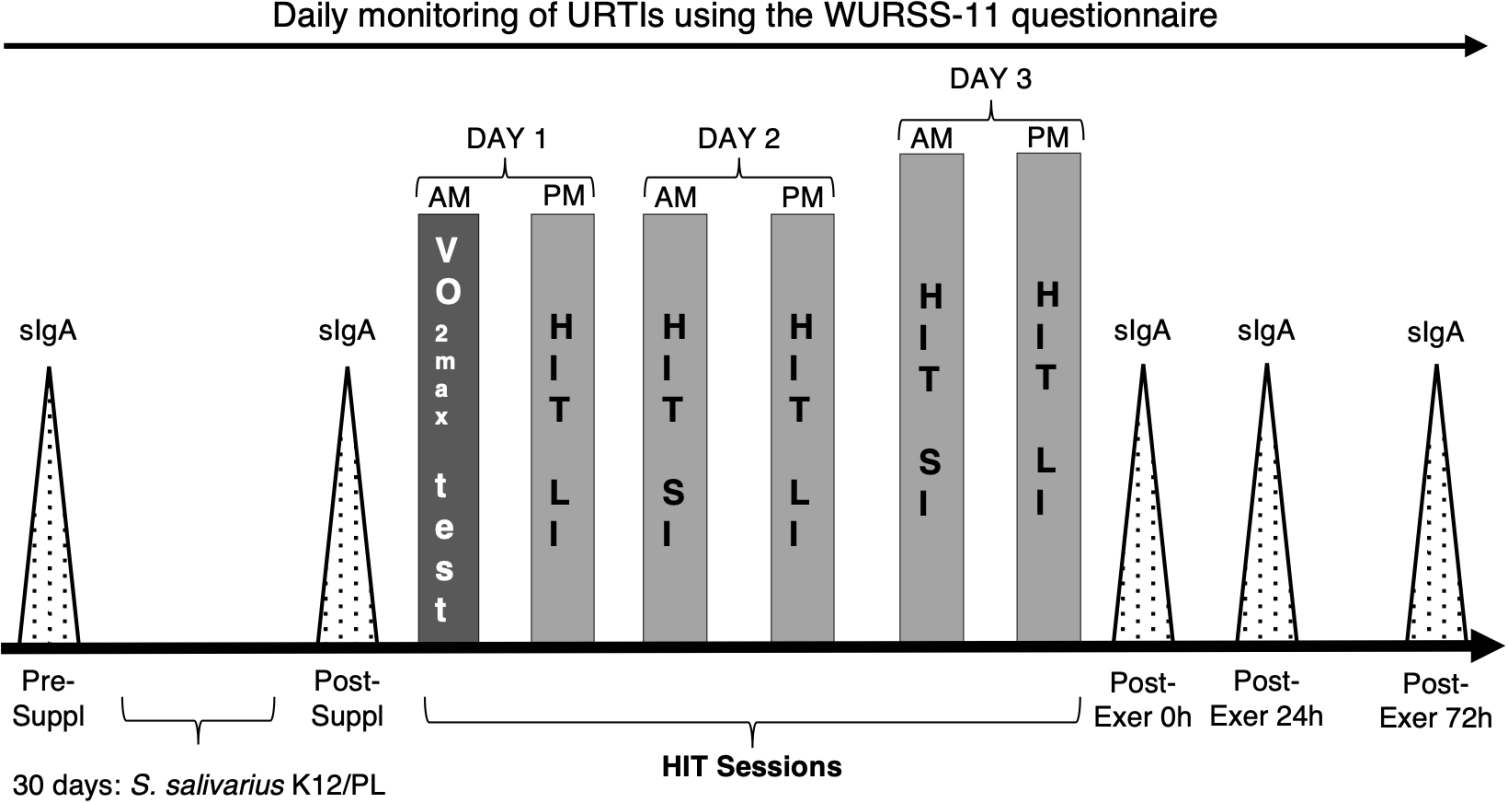
Experimental design. *S. salivarius* K12: *S. salivarius* supplement; PL: placebo compound; sIgA: salivary test sessions of immunoglobulin A; Pre-Suppl: salivary tests before *S. salivarius* K12/PL supplementation; 30 days: *S. salivarius* K12/PL: period of *S. salivarius* K12 or PL intake; Post-Suppl: salivary tests after *S. salivarius* K12 or PL intake; Post-Exer 0h-24h and 72h: salivary tests immediately after training sessions, after 24 and after 72h. AM, morning session; PM afternoon session. VO_2max_ test session; HIT LI: long intervals of high-intensity training; HIT SI: short intervals of high-intensity training.

The participants were asked to take either a preparation containing the probiotic *S. salivarius* K12 (Bactoblis^®^, PharmExtracta S.p.A., Pontenure, Italy) or a placebo with the same characteristics in terms of consistency, taste, and smell. Participants were randomised using block randomisation with blocks of n = 4. The participants and staff enrolling patients were all blinded to treatment conditions. The membership was randomly assigned using a computer-generated random sequence. The compound was taken daily, starting 30 days before the phase including a graded exercise test (GXT) and HIT sessions, and throughout the protocol. Participants were asked to take the compound in the evening as a last gesture before going to sleep. In addition, participants were asked each evening to complete the Wisconsin Upper Respiratory Symptom Survey (WURSS-11) to monitor physical activity levels and health status, particularly the possible presence of URTI (30). URTI episodes were recorded through the 30 days of *S. salivarius* K12 supplementation, the GXT and HIT sessions, and one week after the last training session. URTI episodes were defined by answering “Yes” to the question “Do you think you have a cold?” and scoring at least 2 points on the Jackson scale, which sums the scores of eight cold symptoms, rated on a Likert scale ranging from 0 = absent to 3 = severe. Daily physical activity was monitored by asking the participant if they trained that day, and if yes, the participant indicated which sport they practised, if it was outdoor or indoor, the duration of the training session in minutes, and the perceived intensity of the whole session (on a Borg CR-10 scale). An operator reminded participants to take the product and complete the online questionnaire. On the 31st day after the start of intake, participants were invited to the laboratories in an overnight fasting state (between 8:00 and 9:00) to proceed with the start of the GXT and HIT sessions phase. The procedure involved performing the first salivary sampling, which was immediately followed by the execution of a maximal GXT to assess maximum oxygen consumption and power at the anaerobic threshold. Then all participants were recalled in the afternoon of the same day to begin HIT sessions, as described in **Table 1** and **Figure 1**. The baseline assessment of the abundance of the genus *Streptococcus* in the subjects evaluated was not considered necessary since the literature already describes the colonising capacity of *S. salivarius* K12 both in adults (27) and children (28), against group A Streptococci (such as pyogenes) and group B Streptococci (such as agalactiae) (31). It is also reported a superiority in competitive antagonism compared to other streptococci due to the known production of salivaricins and to the adhesion capacity (32), mechanisms which overall, together with a blocking capacity of adhesion sites, were also shown to be effective against *Streptococcus pneumoniae* (33).

**Table 1:** High-intensity interval training schedule.

**Day 1, AM**	VO_2max_ test
**Day 1, PM**	45’ HIT long intervals 10’WU 6 x 3’(90%VT_2_) and 2’(40%VO_2max_) 5’CD
**Day 2, AM**	45’ HIT short intervals 10’WU 10 x 1’(120%VT_2_) and 2’(30%VO_2max_) 5’CD
**Day 2, PM**	45’ HIT long intervals 10’WU 6 x 3’(100%VT_2_) and 2’(40%VO_2max_) 5’CD
**Day 3, AM**	60’ HIT short intervals 10’WU 10 x 1’(130%VT_2_) and 2’(30%VO_2max_) + 5’ (50%VO_2max_) + 5 x 30” (150%VT_2_) and 1’30” (40%VO_2max_) 5’CD
**Day 3, PM**	60’ HIT long intervals 10’WU 6 x 3’(90%VT_2_) and 2’(40%VO_2max_) + 5’ (50%VO_2max_) + 2 x 4’ (100%VT_2_) and 2’ (40%VO_2max_) 5’CD

WU, warm-up; CD, cool-down.

### 2.3 Product characteristics

The testing product (Bactoblis^®^), in accordance with Italian law no. 169/2004, was notified to the Minister of Health in July 2011 (registration no. 53435) and registered as a food supplement. It contains, at the time of manufacturing, 5 billion CFU/tablet of *S. salivarius* K12 ATCC BAA-1 024 (BLIS Technologies Ltd., New Zealand) and has been manufactured by S.I.I.T. (Trezzano sul Naviglio, Milan, Italy) in the form of a round-shaped, vanilla-flavoured, slowly dissolving tablet. The placebo product was realised in the same form, colour, consistency, solving time, and flavour but with the complete absence of probiotic bacteria.

### 2.4 Saliva samples collection and analysis

Saliva samples were collected in the morning (between 8:00 and 9:00), at the following moments: before and after 30 days of *S. salivarius* K12 administration, and immediately, 24h and 72h after the last exercise training session. Saliva samples were taken by the oral fluid collector (OFC) swab (Soma Bioscience, Wallingford, United Kingdom) following the manufacturer’s instructions (34). Briefly, the OFC swab was put on the top of the tongue until the indicator on the stem turned blue (typically after 1-4 min). During sampling, participants were asked to perform minimal orofacial movement and not suck the swab. The OFC swab collected 0.5 ml of saliva. The whole swab was then placed in the bottle containing the OFC buffer solution and gently mixed by rotation for 10 min at room temperature using a hybridisation oven/shaker (Amersham Biosciences Europe GmbH, Milan, Italy). Three drops of the saliva/buffer mix were added to the sample window of the SOMA sIgA & Cortisol lateral flow device (LFD) and incubated at room temperature for 15 min. The LFD was scanned using the SOMA Cube reader. The sIgA secretion rate was determined by multiplying the saliva flow (0.5 mL × min^-1^) by the sIgA concentration (µg × mL^-1^). All saliva samples were collected after an overnight fast, and participants were asked not to chew gum or brush their teeth before testing to reduce saliva measurement error. Furthermore, volunteers were prohibited from consuming alcoholic beverages or caffeine for 24 hours before saliva collection.

### 2.5 Graded exercise testing procedures

After taking the compound for 30 days and immediately after collecting saliva samples, participants performed a GXT to obtain the power value at maximum oxygen consumption (VO_2max_) and power value at the Ventilatory Threshold (VT_2_). These parameters were used to plan the training sessions using the HIT method. Specifically, the participants used the same cycle ergometer for testing and training sessions (Skillbike, Technogym SpA, Cesena, Italy). The GXT was performed with a ramped procedure in increments of 25W min^-1^ until exhaustion immediately after a conventional 10-minute warm-up at 50W, according to Pallares et al. (35). Polar H10 sensors (Polar Electro Oy, Kempele, Finland) recorded participants’ heart rates during the GXT. Each participant indicated their rate of perceived exertion every minute using the CR10 Scale 0–10, where 0 is defined as no exertion at all, and 10 as “maximum, strenuous” effort [according to Foster et al. (36)]. The peak power value of the last step of GXT was used to derive the values of VO_2max_ and power at VT_2_.

### 2.6 Training protocol and training monitoring

The participants performed six sessions in three days, where the first session was the GXT. The anti-meridian (AM) sessions start between 08:30 AM and 01:00 post-meridian (PM), while the PM sessions start between 02:00 and 07:00 PM. The HIT sessions were designed with the aim of overexercising participants who had no experience with the HIT method or the twice-daily sessions. Each HIT session was performed as group cycling training (5 members per group) by projecting the day’s session schedule on the wall in real-time. Specifically, we planned two formats: short intervals, alternating intervals with power above threshold (110-150%), and recovery intervals at intensities between 30 and 40% VO_2max_ power. On the other hand, long intervals involved intervals between 90-100% of threshold alternating with recovery intervals at intensities between 40-50% of power at VO_2max_. Each session began with a warm-up phase and ended with a cool-down phase. In addition, the time of the sessions was increased from 45 to 60 minutes (second to third day) by increasing the number of intervals (see **Table 1**). To monitor the participants’ training load, after 30 minutes of each training session, a sports scientist asked each participant to provide him or her with the perceived exertion rate of the entire session (sRPE). The CR10 scale value was multiplied by the minutes of each training session.

### 2.7 Statistical analysis

Descriptive statistics were reported as mean ± standard deviation (SD). Comparisons between GXT data were performed using two-sample t-tests. The effect of *S. salivarius* K12 supplementation, salivary flow, sIgA level and secretion rate was tested using an ANOVA for repeated measures with interaction; dependent variable levels (repeated measures; within-factor) were compared at five time-points: before the supplementation period (pre-suppl), after 30 days of supplementation (post-suppl), and immediately (0h), 24h and 72h after the last HIT session (post-exer 0h, 24h and 72h). Group (treated vs control) was a binary between-factor. Simple contrast analysis (reference category = pre-suppl category) was used to verify if salivary flow, sIgA level and secretion changed between the time points and the groups. Finally, Pearson’s correlation indexes were calculated between flux and sIgA secretion for all measurement times. Due to the small sample size and the sex distribution (14 M/6 F), it was not possible to make any reliable investigation of between-sex differences. All elaborations were performed using SPSS version 22.0 or Excel 365; significance was set at p < 0.05.

## 3 Results

Twenty-four healthy young adults were enrolled for the experimental protocol of the study and were randomly assigned in an equal number to the treated or control group. Twenty of them completed the study (14 males and 6 females), while four abandoned it for personal reasons. The demographic and anthropometric characteristics of the entire group of participants were: age 23.5 ± 2.3 years; height 173.5 ± 10.9 cm; weight 71.9 ± 13.1 kg; BMI: 23.7 ± 2.8 kg/m^2^. All subjects took the *S. salivarius* K12 or a placebo for 30 days, with an adherence rate of 94% (87-100%). At the end of the supplementation period, they underwent three days of acute exercise, comprising a GXT and five HIT sessions.

### 3.1 Graded exercise test and training protocol

Results of the GXT are reported in **Table 2**. No differences were detected in the recorded variables between the treated and control groups.

**Table 2:** GXT results (data are expressed as mean ± SD).

Variables	Treated (n=10)	Control (n=10)	p (t)
Peak Power (watt)	244.5 ± 63.9	252.3 ± 62.6	0.267
HR_max_ (bpm)	185.4 ± 11.1	188.0 ± 7.6	0.550
Threshold power (watt)	210.8 ± 63.9	208.1 ± 44.1	0.914
Threshold HR (bpm)	180.0 ± 10.2	181.7 ± 7.2	0.673
VO_2max_ (ml·kg^-1^·min^-1^)	43.4 ± 8.3	46.4 ± 5.7	0.372

During the HIT sessions, the mean power was 65 ± 5% of the threshold power, and the mean heart rate was 84 ± 4% of the maximal heart rate. However, it should be noted that the mean data of the sessions also include warm-up, cool-down, and recovery phases between high-intensity intervals, so this summary statistic does not reflect the intensity of the training sessions. The mean RPE values were 7.5 ± 1.2 AU, indicating the overall effort perceived by the participants. Overall, the total accumulated training load, computed as session RPE, was 2280 ± 249 AU for the control group and 2260 ± 376 AU in the treated group; the mean training load was similar between groups (p > 0.05).

### 3.2 Salivary flow rate, sIgA level and secretion

**Figure 2:**
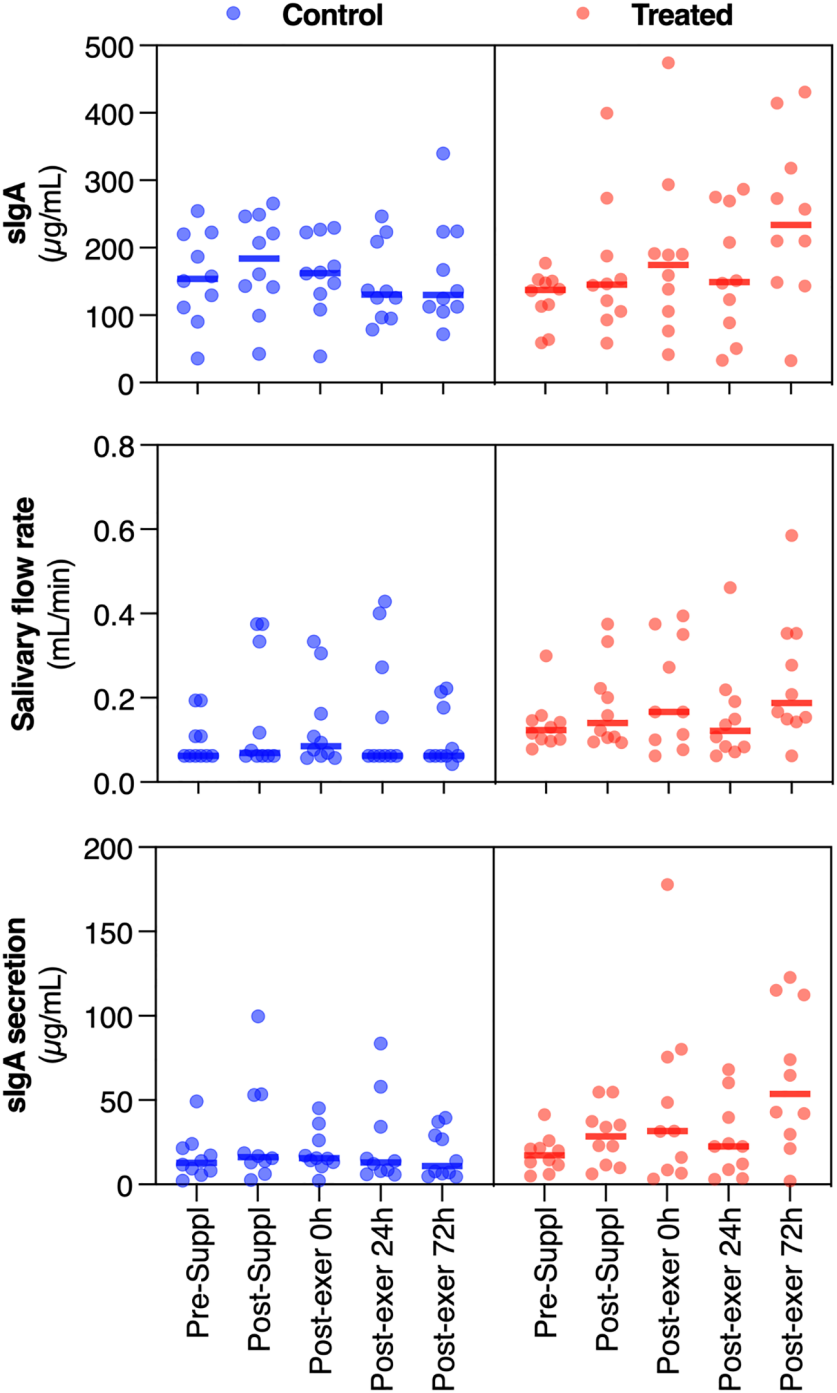
Dot plots of the measured variables (sIgA, salivary flow rate and sIgA secretion), for the treated and placebo groups, at the different time-points (in abscissa axis). The thick line in each set of points represents the median value. Orange dots are about the treated group, while blue dots are about the control group.

Salivary flow rate, sIgA level and secretion at pre-suppl, post-suppl and post-exer 0h, 24h and 72h after the last training session are reported in **Figure 2**.

Considering salivary flux, repeated measures ANOVA revealed non-significant overall time (F_(4,15)_=2.41; p=0.09; $\eta_{\text{p}}^{2}$ =0.391), while time×group (F_(4,15)_ = 3.81; p = 0.025; $\eta_{\text{p}}^{2}$ = 0.504) was significant. In particular, simple contrasts show that salivary flow remains approximately constant across groups except at post-exercise 72h, whose value was significantly higher in the treated group than in the control group.

**Figure 3:**
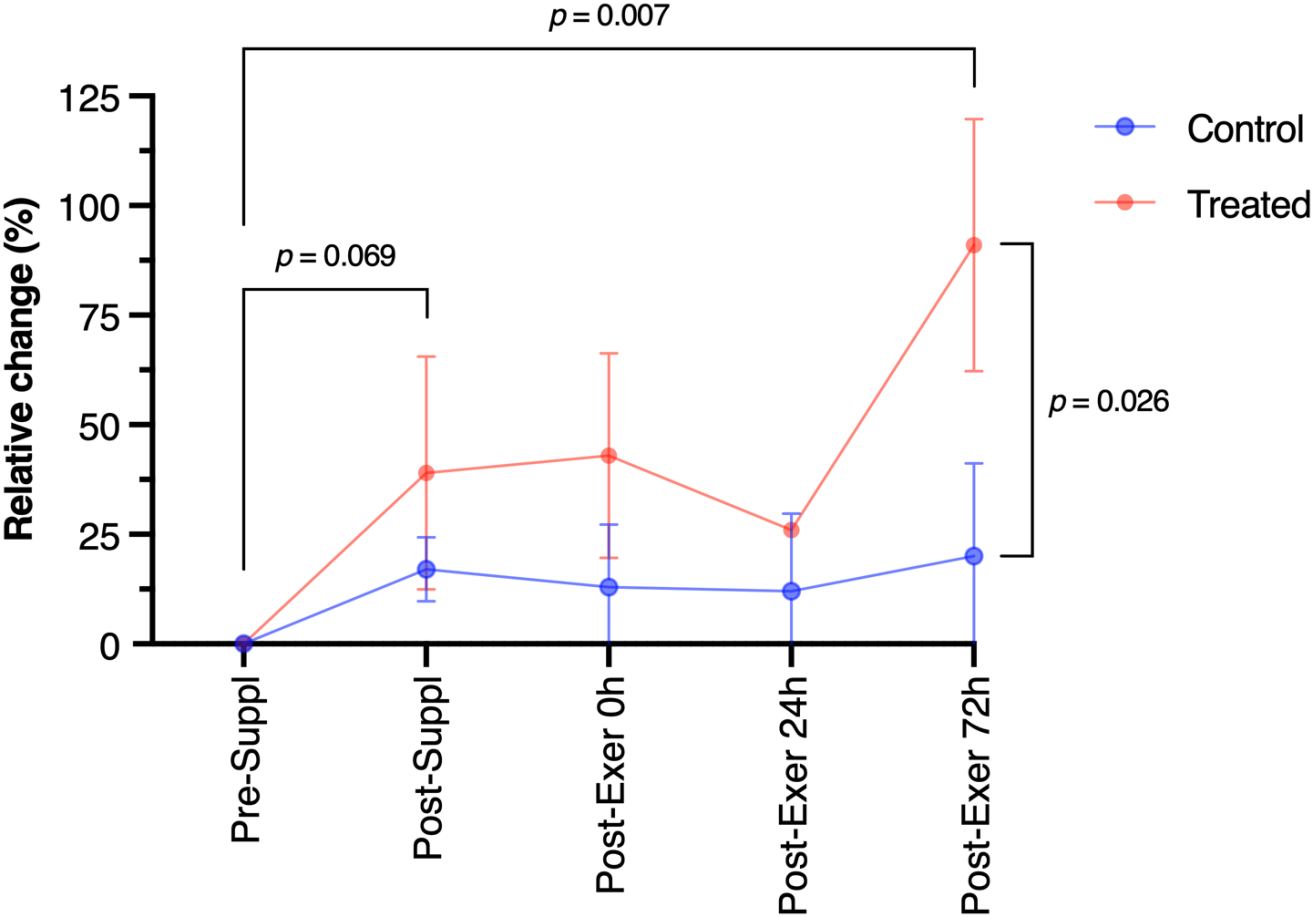
Immunoglobulin A (sIgA) relative change along the study period. Phase refers to before the supplementation period (pre-suppl), after 30 days of supplementation (post-suppl), immediately after the last training session (post-exer 0h), and the remaining time-points refer respectively to 24h and 72h after the last training session (post-exer 24h and 72h). Orange dots are about the treated group, while blue dots are about the control group.

Considering sIgA, repeated measures ANOVA revealed significant time (F_(4,15)_ = 3.38; p = 0.037; $\eta_{\text{p}}^{2}$ = 0.474) and time×group (F_(4,15)_ = 2.49; p = 0.049; $\eta_{\text{p}}^{2}$ = 0.122) effects. Simple contrasts analysis showed a quasi-significant increment of sIgA in the post-suppl (30 days) condition (p = 0.069) with respect to the baseline value, while a significant increase can be noted in the post-exercise 72h condition (p = 0.007). Moreover, in the post-exercise 72h condition, the treated group showed significantly higher IgA levels (p = 0.026). Relative changes (% of variation) of sIgA with respect to pre-supplementation values are reported in **Figure 3**.

Finally, considering sIgA secretion, repeated measures ANOVA revealed significant time (F_(4,15)_ = 6.00; p = 0.004; $\eta_{\text{p}}^{2}$ = 0.615) and time×group (F_(4,15)_ = 5.01; p = 0.009; $\eta_{\text{p}}^{2}$ = 0.572) effects. Simple contrasts revealed an increase in sIgA secretion in the treated group at 0 and 72 hours after exercise. Pearson’s R values considering between flux and IgA secretion variables were, for all times tested, >0.8, showing a significant correlation between variables.

### 3.3 URTI episodes

URTI episodes were recorded using the WURSS-11 questionnaire. The number of episodes in the treated and control groups is reported in **Table 3**. The Chi-square test did not reveal significant differences between the groups (χ² = 1.83; p > 0.05). The total accumulated training load (sRPE) in the supplementation period was roughly the same in the two groups (treated: 4345 ± 3441 AU; control: 4969 ± 4165; p > 0.05).

**Table 3:** Number of days without and with a URTI episode in the supplementation period.

	No (%)	Yes (%)
**Control**	189 (96%)	8 (4%)
**Treated**	214 (98%)	4 (2%)

## 4 Discussion

This study aimed to investigate if 30 days of prophylactic administration of *S. salivarius* K12 could support the mucosal immune function of active young subjects performing HIT workouts. The main results of this study show a slight increase in sIgA level after the *S. salivarius* K12 supplementation and increased sIgA level and secretion rate and salivary flow in the 0-72 h post HIT compared to the placebo group. The number of URTI episodes remained low throughout the study in both groups.

The human oral cavity contains over 700 bacterial species and represents the second most complex microbiota in the human body (37). Much evidence points to an association between bacterial pathogens and oral diseases, while little attention was paid to potentially beneficial bacterial species in the oral cavity (38). *S. salivarius* K12, first isolated from a child with healthy oral tissues, has gained interest in paediatric care due to its effectiveness in reducing recurrent streptococcal upper respiratory tract infections (25, 39–44). Moreover, in adults with recurrent oral streptococcal pathology, 90 days of *S. salivarius* K12 administration reduced pharyngeal tonsillitis by about 80% throughout the study and by 60% in the six months following the use of the probiotic (18). Recent evidence suggests that the supply of *S. salivarius* K12 may also be effective against viral infections (40, 42, 45, 46), a finding which is also relevant in the outbreak of the COVID-19 pandemic.

URTIs are the most common illnesses affecting athletes and can cause absences from training and competition (10, 11). Athletes showed an increased incidence of URTI during periods of intense training or competition (12, 13). Several risk factors and biomarkers associated with increased URTI episodes have been identified (47). sIgA is the most studied immune parameter, and there is a strong negative correlation between the incidence of URTI and sIgA levels in athletes (46). Despite the consistent association between lower concentrations of sIgA and an increased incidence of URTI, the effect of high-intensity exercise on sIgA levels has shown conflicting results. Some studies showed a transient decrease in sIgA up to 24 hours after strenuous training sessions or competitions (6, 46, 48–51), while others were unable to confirm such findings (52–54). A recent meta-analysis by Drummond and colleagues (46) demonstrated increased levels of sIgA after acute exercise, but only in trained subjects. In the present study, at the end of the 30 days training program, the sIgA level slightly increased in both the treated and control groups, although this improvement was greater in the *S. salivarius* K12 supplemented subjects. Notably, the subjects in this study were exercise trained and were able to maintain their habitual physical activity patterns throughout the experimental period. Accordingly, during the 30 days of supplementation, we recorded only eight and four mild-severity URTI events in the control and treated groups, respectively. Interestingly, Babina et al. (17) recently reported that 4 weeks of *S. salivarius* K12 supplementation did not affect the sIgA production, secretion, and salivary flux in healthy subjects. The different modality of *S. salivarius* K12 supplementation between our study and that of Babina et al. (17), which included 2- weeks of probiotic washout, might at least partially explain the divergent results. Moreover, all subjects recruited in the present study were moderately trained and continued their recreational physical activities during the *S. salivarius* K12 supplementation period. As a result, the effect of *S. salivarius* K12 supplementation on sIgA may be influenced by training status. Another possibility is that *S. salivarius* K12 supplementation might have an adjuvant effect on sIgA, fostering a synergic effect with exercise. This hypothesis is supported by the increased sIgA level observed in the *S. salivarius* K12-treated group during the HIT sessions, as described subsequently. Several studies suggest that the risk of URTI can be reduced with moderate stress provided chronically, delineating a J-shaped load-URTI relationship where both insufficient and excessive activity can contribute to the increase in the risk of infection (11, 55, 56). Interestingly, Leandro et al. (57) showed that exercise intensity thresholds correlated to an immune-depressive effect with reduced production of sIgA in duration >2 h and/or intensity >80% of maximal oxygen uptake. Along this line, in this study, we also analysed the effect of six high-intensity exercise sessions performed on three consecutive days to simulate a high training load typical of a training programme of recreational and elite athletes. Here, we found that the sIgA level and secretion did not change immediately and until 72 hours after the acute exercise sessions, despite the significant training load, confirmed by the high session RPE scores. Moreover, URTI episodes did not increase within one week after the high training load exposure. Thus, our results did not support the hypothesis of general transient sIgA reduction and immunosuppression after high-intensity exercises or increased training loads in recreationally active individuals. Interestingly, we also found that the *S. salivarius* K12-treated group showed increased sIgA levels after 30 days of probiotic supplementation, immediately after the HIT sessions, and until 72 h post-exercise. Moreover, both sIgA secretion and salivary flux increased in the treatment group in the post-exercise period compared to the control group. Taken together, our results agree with recent evidence showing that mucosal immune function is a highly adaptable system able to respond quickly to stress, including intensified training load (52). Moreover, the controlled indoor environment of the gym during the HIT sessions might have reduced the immunosuppression risk since it is known that unfavourable ambient conditions (e.g., excessive cold or heat, humidity, and exposure to air pollutants) affect local and systemic immune responses independently from the exercise load (52).

HIT exercise sessions are commonly introduced during a training programme to maximise central and peripheral adaptation in recreational and elite athletes (52). However, in subjects with low sIgA levels and secretion, the increased training load within a short period of time might increase the URTI risk. For example, a polarised training approach based on continuous and interval training sessions increased the URTI events in recreational male endurance runners but only in those with low basal sIgA levels (58). Moreover, sIgA concentration before the training intervention strongly correlated with the number of sick days during the following 12-week training period (58). In this scenario, the ability of *S. salivarius* K12 to boost the sIgA level and secretion in healthy trained subjects may represent an intriguing and interesting finding. The increased salivary flow rate observed 72h after HIT sessions in the treated group might also be relevant for mucosal immune function since a higher salivary flow is associated with greater buffering capacity, salivary rinse (elimination and dilution of undesirable components) and enhancement of the antimicrobial action (59).

The mechanisms explaining the effects of *S. salivarius* K12 administration on mucosal IgA have been recently described in the context of SARS-CoV infection (60, 61). Interestingly, *S. salivarius* K12 administration increased the IgA production against the SARS-CoV-2 Spike protein RBD in mice. Moreover, patients with severe COVID-19 showed a reduction of mucosal anti-RBD IgA. Accordingly, a randomised controlled trial recently demonstrated that the *S. salivarius* K12 supplementation to hospitalised COVID-19 patients improved the main disease markers (60). Possible mechanisms responsible for these effects include the induction of tonic type-I IFN responses (62) and the regulation of systemic and mucosal TGF-β1 levels, with TGF-β1 as an inducer of class switch recombination antibodies in IgA (63, 64). These data outline a picture consistent with what was observed in our study. Hence, the precision probiotic approach with *S. salivarius* K12 seems a promising candidate to increase sIgA level and optimise mucosal microbial colonisation and mucosal barrier function.

In this study, we demonstrated that relatively short-term *S. salivarius* K12 supplementation increased sIgA level and secretion in healthy subjects performing regular exercise training and a demanding exercise-training programme composed of HIT sessions with short recovery time. The limited number of subjects examined, the use of a single type of training stress, the few URTI episodes recorded during the study and the unbalanced ratio of genders constitute the work’s limitations. Future studies on larger samples over longer durations and the use of other types of training stresses (managed by intensity and duration) could provide a more complete view of the potential benefits of *S. salivarius* K12 supplementation on URTI incidence.

## Data availability statement

The raw data supporting the conclusions of this article will be made available by the authors, without undue reservation.

## Ethics statement

The studies involving human participants were reviewed and approved by Ethics Committee for Human Experimentation of Urbino University Carlo Bo (no. of approval 29_2020). The patients/participants provided their written informed consent to participate in this study.

## Author contributions

AB, MG, GA, and DS conceived and drew the experiment, MG and GA performed the data collection, MR, DS, and SA performed the statistical analysis, AB, MG, and GA contributed to the discussion, FP and MR revised critically the manuscript. AB, MG, GA, DS, and SA wrote the manuscript. All authors contributed to the article and approved the submitted version.
